# Childhood socioeconomic position and later-life mortality, morbidity and self-rated health: a linked study from the Historical Population Register of Norway and the Tromsø Study 1950–2022

**DOI:** 10.1177/14034948251365024

**Published:** 2025-08-20

**Authors:** Petja L. Langholz, Hilde L. Sommerseth, Doris T. Kristoffersen, Laila A. Hopstock

**Affiliations:** 1 Department of Archaeology, History, Religious Studies and Theology, UiT The Arctic University of Norway, Tromsø, Norway; 2 Department of Health and Care Sciences, UiT The Arctic University of Norway, Tromsø, Norway

**Keywords:** Childhood socioeconomic position (CSEP), life course, adult health, mortality, inequalities, Norway

## Abstract

**Aims:**

Previous studies on childhood socioeconomic position (CSEP) and health have mostly focused on outcomes in early to mid-life or relied on recalled CSEP in adulthood. The objective of this study was to investigate the association of prospectively measured CSEP with a variety of health outcomes in mid to old age among residents in Northern Norway.

**Methods:**

This study linked health data from the population-based Tromsø Study to the Historical Population Register of Norway. Using Cox proportional hazards models, logistic and ordinal logistic regression, we investigated sex-specific associations between fathers’ occupation in 1950 and all-cause mortality (*n*=7056), as well as chronic disease prevalence and self-rated health (*n*=4576) at age 50 years and older among Tromsø Study participants born in 1930–1955.

**Results:**

Self-rated health showed the strongest association with CSEP and a clear social gradient that was more pronounced among women. We found only minor differences in all-cause mortality and varying patterns for prevalence of chronic diseases by CSEP. High CSEP was associated with lower prevalence of chronic respiratory diseases for both women and men, and higher odds for cancer among women. Associations were attenuated when educational level was included in the models.

**Conclusions:**

**Prospectively measured CSEP was associated with later-life health, although to a varying degree depending on the health indicator under study. This study highlights that, beyond binary disease endpoints, a broad indicator such as self-rated health can be an important tool to uncover health inequalities by CSEP in later life, as it summarises a multitude of possible dimensions of health and wellbeing throughout the life course.**

## Background

Research on the persistence of health inequalities increasingly focuses on the transmission of socioeconomic and behavioural risk factors across generations, and how early-life factors can impact health much later in life [1–3]. People who experience adverse social circumstances during childhood are more likely to accumulate further risks throughout their lives. This includes a short educational period, manual labour, living in deprived neighbourhoods, and lifestyle factors such as smoking and high alcohol intake [4]. Beyond the impact on later-life socioeconomic position (SEP) and health behaviours, childhood socioeconomic position (CSEP) is hypothesised to trigger different trajectories of psychological maturation and physiological development depending on exposure to favourable or unfavourable physical and psychosocial environments in early life [2].

Few longitudinal health studies apply record linkage to historical databases [3] or were started early enough to cover the entire life course of the currently older population [5]. Hence, research about early-life predictors of health inequalities is often limited to outcomes spanning from childhood to mid-life. Studies of health disparities at advanced ages have mostly depended on retrospective self-report of childhood conditions [6–9]. How individuals recall these conditions in later life could be influenced by both adult SEP and adult health status [2]. Although some studies have concluded that recalled CSEP is reliable [10], it is nevertheless desirable to utilise prospectively measured exposures whenever possible.

This study is the first to utilise linked data from the population-based Tromsø Study and the Historical Population Register of Norway (HPR). The aim is to investigate the association of prospectively measured CSEP with all-cause mortality, chronic disease prevalence, and self-rated health in mid to old age in two population samples in Northern Norway. We included a variety of health outcomes to assess potential variations in associations by type of indicator.

## Methods

The Tromsø Study is a population-based health study consisting of seven surveys conducted between 1974 and 2016 in Tromsø municipality, Norway. Total birth cohorts and random samples of inhabitants were invited, and more than 45,000 women and men participated in one or more surveys (attendance 65–79%). Data collection consisted of questionnaires, biological samples, and clinical examinations [11]. Unique identification numbers from the National Population Register allowed for complete follow-up of Tromsø Study participants in national health registries, including the Cancer Registry of Norway (CRN) and the Norwegian Cause of Death Registry (NCoDR).

The HPR is a national database under construction. The aim is to include records of the approximately ten million people who lived in Norway from 1801 to 1964, the year in which the current National Population Register was established. The register covers multigenerational life course data constructed by linking records across population censuses and church books. HPR provides individual attributes such as birthplace, marital status, and occupations, and time-stamped vital events such as births, marriages, and deaths. Identification numbers for residents were first introduced in 1964, so linkage between the HPR and modern registers or surveys relies on probabilistic linking methods. Identifying variables including name, sex, birth date, and birthplace are used to link census records of the same individual across sources.

This study was approved by the Regional Committee for Medical and Health Research Ethics (reference 61504), the Norwegian Agency for Shared Services in Education and Research (reference 239497), and the National Archives of Norway (reference AVS-23-01-00270). Tromsø Study participants gave written informed consent. Those who withdrew their consent were excluded from this study.

### Sample selection

This study used links between Tromsø Study participants and their fathers in the Norwegian population census of 1950 derived from the HPR. The exposure of interest was CSEP, measured as the father’s occupation in 1950. Thus, we limited the study to Tromsø Study participants born between 1930 and 1955. We excluded participants whose fathers fell outside the age range of 30–65 years in 1950 to avoid misleading occupational information. The proportion of the defined birth cohorts that could be linked to their fathers in the census was 44.2%. As the census is still under transcription, links are expected to be missing at random with regard to the exposure and outcome under study. Naturally, links were also missing for participants whose fathers were not alive or did not reside in Norway in 1950.

We analysed two samples according to outcome at age 50 years and older: (a) all-cause mortality; (b) chronic disease prevalence and self-rated health (Table I). The mortality analysis utilised information from HPR and NCoDR. The sample included all participants from the linked sample who did not die or emigrate before age 50 years (*n*=7056). Analyses of disease prevalence and self-rated health were conducted in a second sample restricted to participants with complete information on these health outcomes (*n*=4576). Sample size did not allow for analyses stratified by year of survey, so we pooled observations and limited the sample to participants from any of the four last surveys between 1994 and 2016 at age 50 years and older. Due to few participants in the oldest ages, we excluded participants older than 80 years. If participants had attended several surveys, the last measurement within the defined age range was selected. For a detailed description of the sample selection process, see Supplemental Figure 1.

**Table I.** Sample characteristics for women and men.

	Total	Women	Men
Sample for mortality follow-up	*N*=7056	*N*=3043	*N*=4013
Year of birth, mean (SD)	1945 (6.2)	1946 (6.3)	1945 (6.2)
Follow-up time in years, median (IQR)	23.9 (8.6)	24.1 (8.6)	23.8 (8.6)
Dead, *n* (%)	1947 (27.6)	655 (21.5)	1292 (32.2)
Childhood socioeconomic position, *n* (%)			
Upper non-manual	246 (3.5)	105 (3.5)	141 (3.5)
Lower non-manual	1324 (18.8)	583 (19.2)	741 (18.5)
Skilled manual	921 (13.1)	408 (13.4)	513 (12.8)
Farmers	1247 (17.7)	513 (16.9)	734 (18.3)
Unskilled/lower-skilled manual	3318 (47.0)	1434 (47.1)	1884 (47.0)
Sample for chronic disease prevalence and self-rated health	*N*=4576	*N*=2069	*N*=2507
Age at participation, mean (SD)	66.5 (6.2)	66.7 (6.0)	66.4 (6.4)
Year of birth, mean (SD)	1944 (6.4)	1945 (6.4)	1944 (6.4)
Childhood socioeconomic position, *n* (%)			
Upper non-manual	135 (3.0)	60 (2.9)	75 (3.0)
Lower non-manual	827 (18.1)	387 (18.7)	440 (17.6)
Skilled manual	586 (12.8)	279 (13.5)	307 (12.3)
Farmers	835 (18.3)	358 (17.3)	477 (19.0)
Unskilled/lower-skilled manual	2193 (47.9)	985 (47.6)	1208 (48.2)
Disease prevalence, *n* (%)			
Cardiovascular disease	546 (11.9)	134 (6.5)	412 (16.4)
Cancer	548 (12.0)	225 (10.9)	323 (12.9)
Diabetes	494 (10.8)	192 (9.3)	302 (12.1)
Chronic respiratory disease	756 (16.5)	392 (19.0)	364 (14.5)
Self-rated health, *n* (%)			
Bad	259 (5.7)	117 (5.7)	142 (5.7)
Neither good nor bad	1543 (33.7)	721 (34.9)	822 (32.8)
Good	2333 (51.0)	1018 (49.2)	1315 (52.5)
Very good	441 (9.6)	213 (10.3)	228 (9.1)

Source: The Tromsø Study 1974–2022; occupational data from the Historical Population Register of Norway (original sources at the National Archives of Norway).

### Childhood socioeconomic position

CSEP was operationalised as the father’s occupation in the census of 1950. The census contains handwritten occupations and corresponding numerical codes. For this study, the codes were translated to HISCLASS, the Historical International Social Class Scheme [12]. Despite their differences, historical and contemporary class schemes are highly correlated when using broad categories [13]. Considering sample size and the available level of detail, the 12 HISCLASS categories were collapsed into the following: upper non-manual workers (HISCLASS 1–2), lower non-manual workers (3–5), skilled manual workers (6–7), farmers (8), and lower-skilled or unskilled manual workers (9–12). Census instructions included the registration of an individual’s ‘usual’ occupation in case of unemployment. Supplemental Table I shows how the father’s occupational class corresponded with two self-reported socioeconomic variables from the Tromsø Study: highest educational level and childhood financial conditions (CFC).

### Outcome variables

The date of death was ascertained through linkage to the NCoDR. This enabled complete follow-up for all-cause mortality until the end of 2022. We assessed previous and/or current disease prevalence of the four major non-communicable diseases (NCDs) from the NCD framework of the World Health Organization (WHO): cardiovascular disease (CVD), cancer, diabetes, and chronic respiratory diseases [14]. CVD was defined as self-reported history of myocardial infarction and/or stroke. Prior or current cancer diagnosis at the time of study participation was retrieved from the CRN. Diabetes was defined as self-reported diabetes and/or haemoglobin A1c (HbA1c) level over 48 mmol/mol based on analysis of blood samples collected at attendance, as described in Langholz et al. [15]. Chronic respiratory disease was defined as self-reported asthma, chronic bronchitis, or chronic obstructive pulmonary disease (COPD). Self-rated health was measured by the following questionnaire item: ‘How do you in general consider your own health to be?’. Response alternatives varied across surveys and were collapsed into the following four categories: ‘very bad’/‘bad’, ‘neither good nor bad’/‘not quite good’, ‘good’, and ‘very good’.

### Statistics

Sample characteristics were summarised as frequencies with proportions for categorical variables and mean or median with standard deviation (SD) or interquartile range (IQR), respectively, for continuous variables (Table I). The association between CSEP and self-reported CFC and education was tested using the chi^2^-test (Supplemental Table I).

All analyses were performed both combined and stratified by sex. We estimated hazard ratios (HRs) and 95% confidence intervals (CIs) using Cox proportional hazards models to assess the association between CSEP and all-cause mortality after age 50 years. Attained age was used as the underlying timescale, and birth year was added as a continuous covariate to account for cohort differences in mortality risk. Participants entered the analysis at age 50 years or thereafter, at first participation in the Tromsø Study. Mortality follow-up lasted until death, emigration, or end of study in December 2022, whichever came first. The proportional hazards assumption was checked by examining the log–log plot and Schoenfeld residuals. We calculated odds ratios (ORs) and 95% CIs using logistic regression to evaluate the association between CSEP and chronic disease prevalence. Due to small numbers, all non-manual occupations were collapsed in these analyses. For the ordinal outcome of self-rated health, we applied ordinal logistic regression to calculate ORs for better self-rated health. All models were adjusted for age and year of survey, which were added as continuous variables. We were primarily interested in capturing the total effect of CSEP on later-life health. Hence, we did not include further covariates that are expected to mediate the association between CSEP and health outcomes in the main analyses. Results for education and CFC as alternative explanatory variables and CSEP adjusted for education are presented in Supplemental Figures 2–5 and Supplemental Tables II–VI. All analyses were carried out in R version 4.2.3. Figures were created using the R package ‘forester’ [16].

## Results

Table I shows characteristics for both study samples stratified by sex. In the mortality sample, the median follow-up time was 24 years. In total, 32.2% of men and 21.5% of women had died until the end of study. Close to half of the participants had fathers in unskilled or lower-skilled work, 18% were farmers and 13% were skilled manual workers. Lower and upper non-manual occupations were held by 19% and 3.5% of fathers, respectively. The occupational distribution was similar in the subsample for disease prevalence and self-rated health. CSEP from 1950 was associated with both self-reported CFC and educational level (Supplemental Table I).

**Figure 1.**
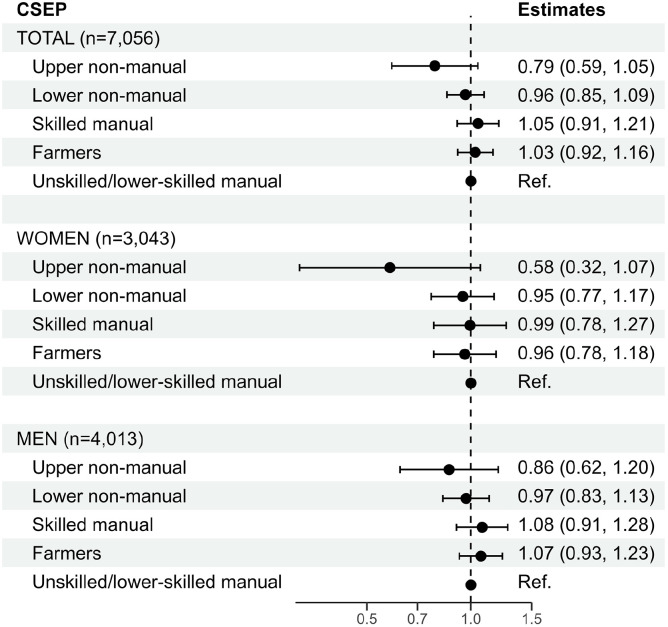
Hazard ratios with 95% confidence intervals from Cox proportional hazards models for all-cause mortality at age 50 years and older by childhood socioeconomic position (CSEP). Hazard ratios adjusted for age (timescale) and year of birth; total sample additionally adjusted for sex. Source: The Tromsø Study 1974–2022; occupational data from the Historical Population Register of Norway (original sources at the National Archives of Norway).

Figure 1 shows hazard ratios for all-cause mortality by CSEP. Effect estimates showed practically no association with mortality, neither for women nor for men. Only for the small group of women with fathers in upper non-manual occupations HRs suggested decreased mortality risk (HR 0.58 (95% CI 0.32, 1.07)) compared with those with fathers in unskilled or lower-skilled work.

Table II presents ORs for chronic disease prevalence by CSEP. Overall, the effect estimates were small, and in many cases the data were compatible with both reduced and increased odds. For CVD, the OR for daughters and sons of non-manual workers was 0.77 (95% CI 0.60, 1.00) compared with lower-skilled and unskilled workers. Results for cancer showed a reverse gradient, especially for women, with 60% higher odds (95% CI 1.11, 2.21) for a cancer diagnosis among women from a non-manual background. Regarding diabetes, the effect estimates did not indicate any social gradient. Instead, the odds were lowest for those with fathers in skilled manual work for both women and men (OR 0.67 (95% CI 0.48, 0.94)). An advantage of high CSEP was most pronounced for chronic respiratory diseases among women and men, with 33% lower odds (95% CI 0.54, 0.84) for the non-manual group.

**Table II.** Odds ratios with 95% confidence intervals from logistic regression models for prevalence of chronic diseases at age 50–80 years by childhood socioeconomic position (CSEP).

	Cardiovascular diseases	Cancer	Diabetes	Chronic respiratory diseases
CSEP				
TOTAL (*n*=4576)				
Non-manual	0.77 (0.60, 1.00)	1.24 (0.99, 1.56)	0.96 (0.75, 1.23)	0.67 (0.54, 0.84)
Skilled manual	0.96 (0.72, 1.29)	1.19 (0.90, 1.57)	0.67 (0.48, 0.94)	0.89 (0.70, 1.14)
Farmers	1.11 (0.88, 1.41)	0.97 (0.75, 1.25)	1.20 (0.94, 1.53)	0.83 (0.67, 1.03)
Unskilled/lower-skilled manual	Ref.	Ref.	Ref.	Ref.
WOMEN (*n*=2069)				
Non-manual	0.77 (0.47, 1.26)	1.60 (1.11, 2.21)	0.78 (0.52, 1.16)	0.68 (0.50, 0.92)
Skilled manual	1.00 (0.58, 1.73)	1.13 (0.73, 1.76)	0.60 (0.35, 1.02)	0.94 (0.67, 1.31)
Farmers	1.05 (0.65, 1.67)	1.16 (0.78, 1.72)	1.19 (0.81, 1.75)	0.94 (0.69, 1.28)
Unskilled/lower-skilled manual	Ref.	Ref.	Ref.	Ref.
MEN (*n*=2507)				
Non-manual	0.77 (0.57, 1.04)	1.04 (0.76, 1.42)	1.10 (0.81, 1.51)	0.67 (0.49, 0.92)
Skilled manual	0.95 (0.67, 1.34)	1.24 (0.86, 1.78)	0.73 (0.47, 1.12)	0.84 (0.59, 1.20)
Farmers	1.13 (0.86, 1.50)	0.85 (0.61, 1.19)	1.20 (0.88, 1.65)	0.73 (0.54, 1.00)
Unskilled/lower-skilled manual	Ref.	Ref.	Ref.	Ref.

Odds ratios adjusted for age and year of survey; total sample additionally adjusted for sex.

Source: The Tromsø Study 1994–2016; occupational data from the Historical Population Register of Norway (original sources at the National Archives of Norway).

**Figure 2.**
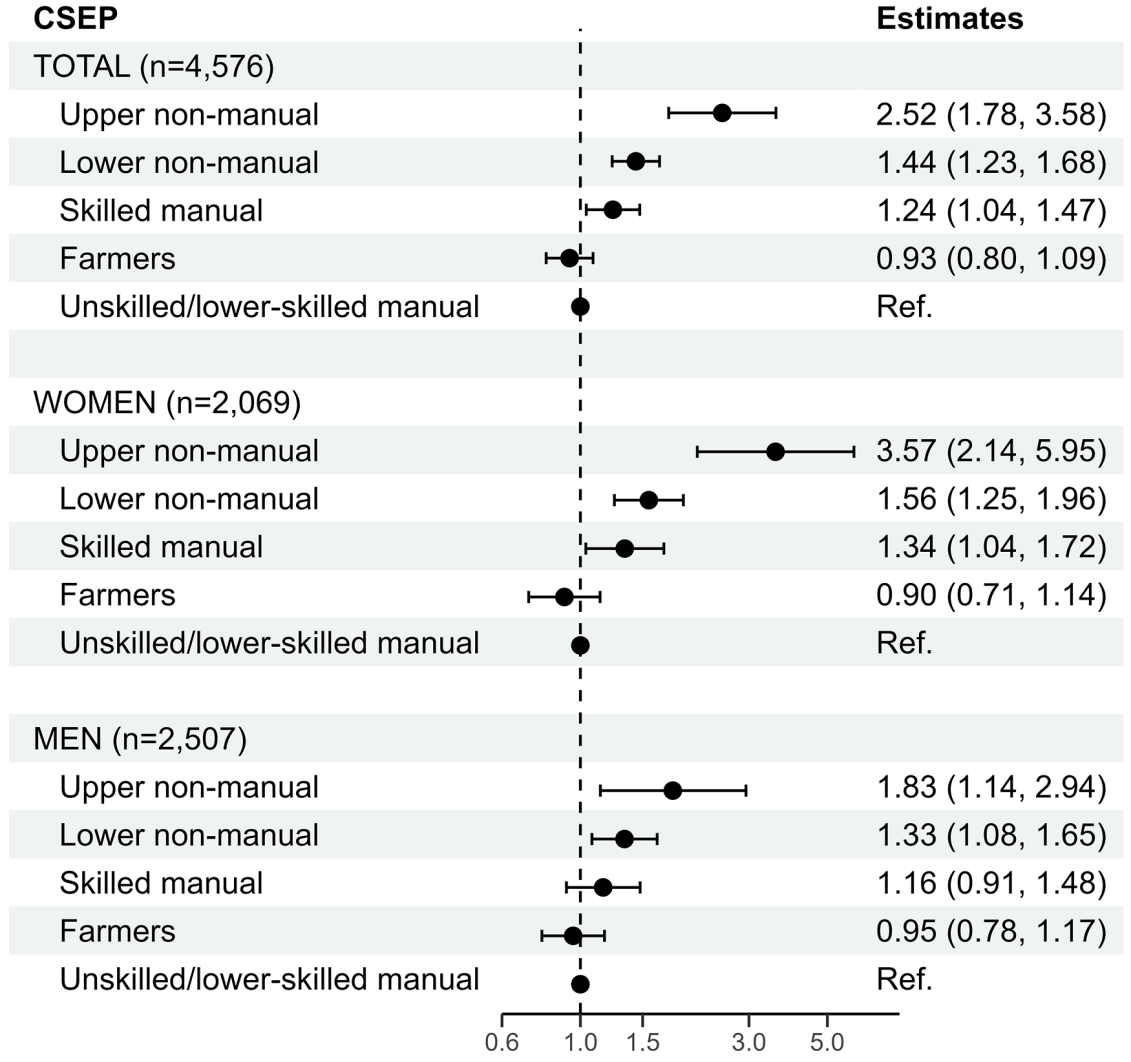
Odds ratios with 95% confidence intervals from ordinal logistic regression models for self-rated health at age 50–80 years by childhood socioeconomic position (CSEP). Odds ratios adjusted for age and year of survey; total sample additionally adjusted for sex. Source: The Tromsø Study 1994–2016; occupational data from the Historical Population Register of Norway (original sources at the National Archives of Norway).

There was a stepwise increase in odds for reporting better self-rated health with increasing CSEP, apart from the farmer category (Figure 2). This social gradient was more pronounced for women. The group of women with fathers in upper non-manual occupations had 3.57 times higher odds for better self-rated health compared with the reference group (95% CI 2.14, 5.95). For men, the respective OR was 1.83 (95% CI 1.14, 2.94).

In supplementary analyses, recalled CFC showed little association with mortality and chronic diseases (Supplemental Figure 3, Supplemental Table III). In contrast, educational level revealed social gradients for all health outcomes (Supplemental Figure 2, Supplemental Table II). Similar to the prospective CSEP measure, both education and CFC presented the largest effect estimates for self-rated health (Supplemental Figures 4 and 5). Where CSEP was associated with later-life health, the effect estimates were attenuated when adjusting for education (Supplemental Tables IV–VI). For self-rated health, only women with fathers in upper non-manual occupations retained significantly higher odds for better self-rated health independent of their own educational level (OR 2.17 (95% CI 1.27, 3.71)) (Supplemental Table VI).

## Discussion

This study investigated associations of prospectively measured CSEP with several health outcomes in later life. We found few differences in all-cause mortality and varying patterns for the prevalence of chronic diseases. Self-rated health showed the strongest association with CSEP and a clear social gradient, especially among women.

Contrary to our findings, systematic reviews have previously presented evidence for associations of CSEP with adult mortality [17], CVD [18], and diabetes [19]. The association between SEP and cancer risk varies depending on the type of cancer. Strong negative social gradients are usually found for lung cancer and other smoking-associated cancers, while positive gradients have been found for prostate and breast cancer, among others [20]. In our study, breast cancer was likely to be driving the positive social gradient observed for women, explained by both increased risk (e.g. nulliparity) and higher detection rates through screening among women with high SEP [20]. Among both women and men, odds for chronic respiratory diseases were significantly lower for those with fathers in non-manual occupations. Besides smoking as the strongest and most obvious mediator in this association, exposure to dusts and fumes at the workplace has been linked to an increased risk of respiratory diseases such as COPD – also among never-smokers [21]. One can assume that participants with fathers in non-manual occupations were less likely to be exposed to these environmental risks at their own workplaces. Social mobility increased considerably throughout the 20th century. Yet, from 1960 to 1980, when our study cohort would have entered the labour market, sons of white-collar workers were still 19 times more likely to enter a white-collar occupation over an unskilled occupation compared with sons of unskilled workers [22].

The clearest social gradient was found for self-rated health, especially among women. Given the dual nature of self-rated health as a ‘subjective and contextual self-assessment’ as well as an ‘indicator of objective somatic and mental state’ [23], one could expect self-rated health to present a stronger association with CSEP than any single disease outcome, as it summarises various dimensions of health and perceived limitations. Based on the modest results for chronic diseases in this study, it seems likely that the results for self-rated health are driven by further health dimensions beyond the major NCDs. An important element of self-rated health in later life is mental health [24], which was shown to be associated with childhood socioeconomic conditions, especially among women [8]. Another possible pathway from CSEP to later-life self-rated health is the intergenerational persistence of occupational careers. For example, lasting inequalities in bodily pain were found between occupational groups across the life course [25]. While we could not assess intergenerational occupational persistence in our study, education – another indicator of adult SEP – attenuated most of the measured associations between CSEP and health outcomes, including self-rated health. This aligns with previous research that identified educational attainment as an important mediator on the pathway between childhood socioeconomic conditions and later-life health [7,9].

Similar to our study, van de Mheen et al. [6] found that the association between CSEP and health differed by the type of indicator in a Dutch population sample aged 25–74 years. Results indicated weak associations with mortality, a stronger gradient for the number of health complaints, and the largest effect estimates for self-rated health [6]. Evidently, mortality or disease diagnosis is not an exhaustive indicator of health, and people are increasingly living with chronic conditions for many years. Day-to-day wellbeing, sense of agency and quality of care can therefore be decisive in perceived health beyond the mere diagnosis. Thus, disparities between socioeconomic groups might be captured especially well through broad measures such as self-rated health.

### Considering period and cohort effects

The mean birth year of participants in this study was 1944–1946. During the Second World War up until the 1950s, Norway witnessed a considerable drop in income inequality, which only started to reverse in the 1980s [26]. The socioeconomic differences captured by occupational groups in the census of 1950 might therefore have been less marked than in earlier or later periods. A study on the implementation of cigarette taxation suggested that reductions in early-life exposure to smoking were associated with a higher age at death for US cohorts born during the 1920s and 1930s. Moreover, smoking exposure in childhood had a greater effect on mortality than parental SEP [27]. Smoking used to be common in higher socioeconomic strata in Norway, and the inverse social gradient in smoking only manifested itself in the second half of the 20th century [28]. Consequently, one can expect that CSEP is more highly correlated with childhood exposure to smoking in recent decades. Hence, differences in mortality, CVD, or respiratory diseases due to early-life exposure to smoking might not be captured well by categories of fathers’ occupation in our study. Whether these cohorts represent generations with weaker health disparities by CSEP compared with earlier or later cohorts could be a valuable topic for future longitudinal studies.

### Strengths and limitations

CSEP was measured at a single, fixed time point in 1950. As occupation often varies over the life course, a single measurement might misrepresent CSEP for some individuals. Moreover, there is variation in income and wealth within occupational groups which our CSEP measure cannot account for. Farmers are a particularly heterogeneous group that is difficult to place in an occupational hierarchy. This study also lacked information on place of birth or residence in 1950. The fathers’ occupation likely captured some spatial information from childhood (e.g. more participants from non-manual occupational backgrounds grew up in metropolitan regions), which might have had an impact on later-life health. Participants were born between 1930 and 1955, a time span with considerable societal and economic changes. The linkage rate for this study restricted the sample size, age range and desirable investigations of cohort differences within the sample. Once transcription and linkage between the HPR and modern sources have progressed further, future studies should aim to assess not only cohort effects but also socioeconomic trajectories across the life course and their effect on later-life health. Furthermore, we assessed self-reported history of CVD, which naturally excludes fatal events. If participants with lower CSEP experienced higher CVD mortality earlier in the life course, we might have underestimated this association.

A strength of this study was that several outcomes were assessed through linkage to national registers (mortality and cancer) and through additional objective measurements (diabetes). CSEP was collected prospectively several decades before study participation, so that concerns regarding differential recall bias [29,30] could be discarded.

## Conclusions

This analysis, based on first-time record linkage between a Norwegian health study and the census of 1950, found that prospectively measured CSEP was associated with later-life health. Yet, the strength of this relationship varied depending on the specific health indicator under study. We found only minor differences in all-cause mortality, while high CSEP was associated with a lower prevalence of chronic respiratory diseases for women and men, and higher cancer prevalence among women. Self-rated health showed the strongest association with CSEP and a clear social gradient. Accounting for educational attainment attenuated these associations, indicating the crucial role of later-life socioeconomic achievements. Among the assessed health outcomes, self-rated health might be especially valuable as a single health indicator to uncover lasting inequalities by CSEP in later life, as it summarises a multitude of possible health dimensions. Continued efforts should be made to expand record linkage between historical and modern sources of population data to facilitate longitudinal studies of period and cohort effects in the relationship between socioeconomic conditions and health over the life course and across generations.

## Supplemental Material

sj-docx-1-sjp-10.1177_14034948251365024 – Supplemental material for Childhood socioeconomic position and later-life mortality, morbidity and self-rated health: a linked study from the Historical Population Register of Norway and the Tromsø Study 1950–2022 Supplemental material, sj-docx-1-sjp-10.1177_14034948251365024 for Childhood socioeconomic position and later-life mortality, morbidity and self-rated health: a linked study from the Historical Population Register of Norway and the Tromsø Study 1950–2022 by Petja L. Langholz, Hilde L. Sommerseth, Doris T. Kristoffersen and Laila A. Hopstock in Scandinavian Journal of Public Health
