# Supplementary material for: Childhood socioeconomic position and later-life mortality, morbidity and self-rated health: a linked study from the Historical Population Register of Norway and the Tromsø Study 1950–2022
Source: Scand J Public Health. 2025 Aug 20;54(6):663–71. doi: 10.1177/14034948251365024 (PMC13356268; doi:10.1177/14034948251365024)
Supplement: sj-docx-1-sjp-10.1177_14034948251365024 – Supplemental material for Childhood socioeconomic position and later-life mortality, morbidity and self-rated health: a linked study from the Historical Population Register of Norway and the Tromsø Study 1950–2022 [file sj-docx-1-sjp-10.1177_14034948251365024.docx]

Excluded: n=39

(missing or unclassifiable occupational data for fathers in the 1950 census)

n=7,188

(3,076 women, 4,112 men)

Excluded: n=1,777

(fathers under 30 or over 65 years old in 1950)

n=7,227

(3,092 women, 4,135 men)

Excluded: n=11,365

(missing linkage to fathers in the 1950 census)

n=9,004

(3,928 women, 5,076 men)

Excluded: n=24,767

(born before 1930 or after 1955)

n=20,369

(9,602 women, 10,767 men)

n=45,136

(22,622 women, 22,514 men)

**Sample for mortality follow-up:**

n=7,056

(3,043 women, 4,013 men)

**Sample for disease prevalence and self-rated health:**

n=4,576

(2,069 women, 2,507 men)

Excluded: n=132

(died or emigrated before age 50)

n=45,473 attended one or more surveys of the Tromsø Study between 1974 and 2016

Excluded: n=337

(withdrawn consent)

Excluded: n=2,480

(no participation in Tromsø4–Tromsø7: n=1,653,

no participation at age 50–80: n=663,

missing data on health outcomes: n=164)

**Supplementary Figure S1**. Flow chart of the study population

Source: The Tromsø Study 1974–2016; occupational data from the Historical Population Register of Norway (original sources at the National Archives of Norway).

**Supplementary Table S1.** Self-reported socioeconomic variables in the Tromsø Study by father’s occupational class in 1950.

|  | **Unskilled/**  **lower-skilled manual** | **Farmers** | **Skilled manual** | **Lower**  **non-manual** | **Upper**  **non-manual** | **P-value** |
| --- | --- | --- | --- | --- | --- | --- |
| **Childhood financial conditions** |  |  |  |  |  | <0.001 |
| Very good | 3.6 (105) | 3.1 (34) | 5.4 (44) | 11.4 (130) | 22.4 (46) |  |
| Good | 59.4 (1,751) | 61.1 (673) | 66.0 (540) | 73.9 (843) | 73.2 (150) |  |
| Very difficult/difficult | 37.0 (1,090) | 35.8 (394) | 28.6 (234) | 14.6 (167) | 4.4 (9) |  |
|  |  |  |  |  |  |  |
| **Highest educational level** |  |  |  |  |  | <0.001 |
| Long tertiary education | 9.0 (282) | 13.5 (158) | 20.7 (180) | 30.8 (380) | 66.7 (152) |  |
| Short tertiary education | 12.0 (376) | 12.3 (144) | 16.3 (142) | 19.4 (240) | 15.8 (36) |  |
| Upper secondary education | 28.7 (900) | 27.3 (320) | 32.8 (285) | 28.7 (354) | 15.4 (35) |  |
| Primary education | 50.3 (1,575) | 46.9 (549) | 30.2 (263) | 21.1 (260) | 2.2 (5) |  |

P-value from Chi^2^-test. Total sample n=7,056; missing data on education: n=420 (variable not included in Tromsø1); missing data on childhood financial conditions: n=846 (variable not included in Tromsø1, Tromsø3 and Tromsø5). Data on childhood financial conditions and highest educational level were collected from questionnaires. In Tromsø2, Tromsø3 and Tromsø5 participants were asked: *“How many years of education have you had?”*. The numerical answers were categorized as follows: primary education (up to 9 years); upper secondary education (10–12 years); short tertiary education (13–15 years); long tertiary education (16 years or more). In Tromsø4, Tromsø6 and Tromsø7 participants were asked, *“What is the highest level of education you have completed?”*. In Tromsø7, the four response alternatives corresponded with the aforementioned categories. For Tromsø4 and Tromsø6, a fifth response category for technical/vocational schooling was collapsed with the category for upper secondary education. Childhood financial conditions was assessed by the question: *“How was your family’s financial situation during your childhood?”*. The response alternatives were: *“very difficult”, “difficult”, “good”* and *“very good”*. We collapsed the first two categories due to low frequency. For participants who attended more than one survey, their last response for each variable was selected for the analysis. Source: The Tromsø Study 1974–2022; occupational data from the Historical Population Register of Norway (original sources at the National Archives of Norway).

**
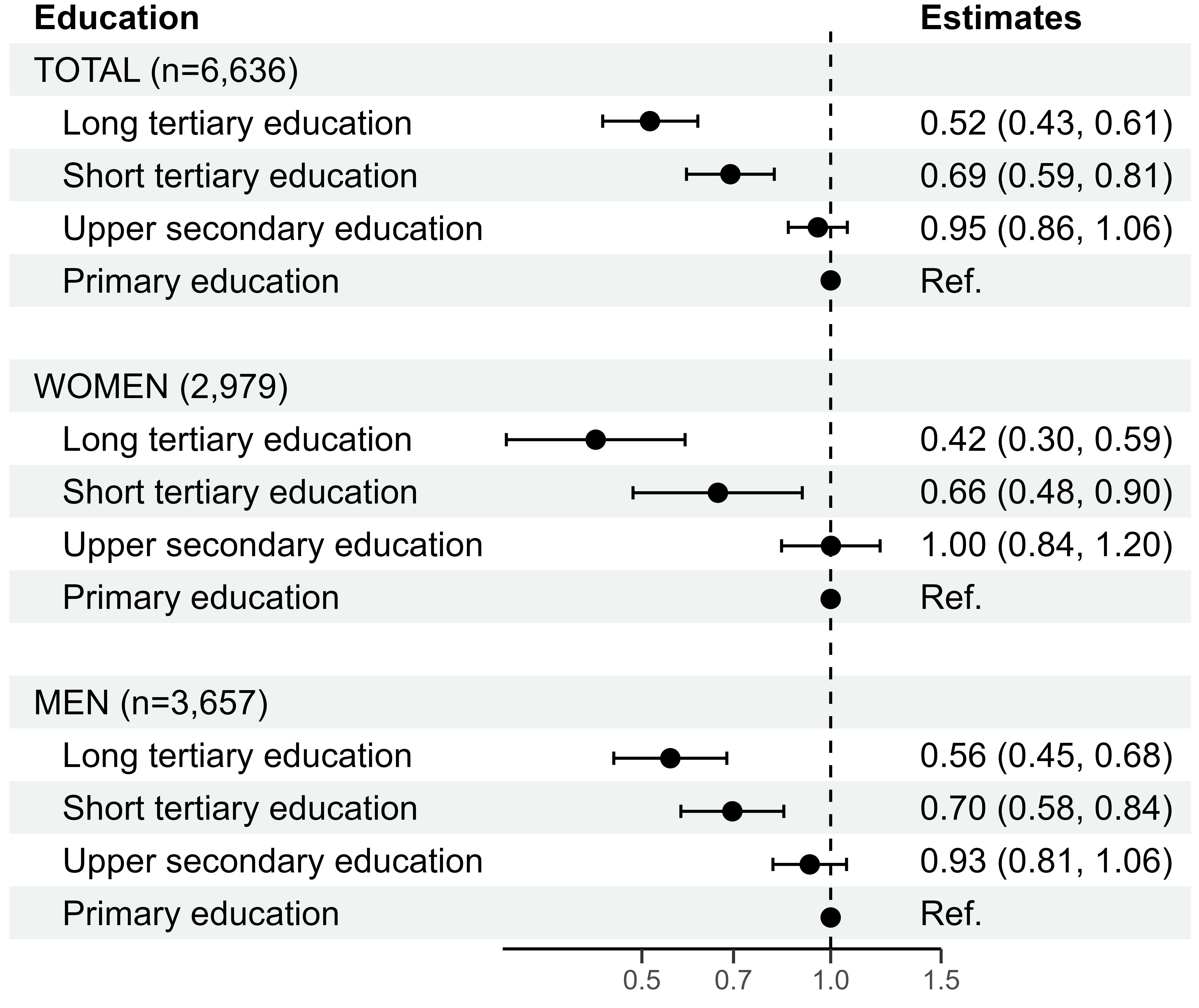
**

**Supplementary Figure S2**. Hazard ratios with 95% confidence intervals from Cox proportional hazards models for all-cause mortality at age 50 and older by highest educational level.

Hazard ratios adjusted for age (timescale) and year of birth; total sample additionally adjusted for sex. Source: The Tromsø Study 1974–2022.

**
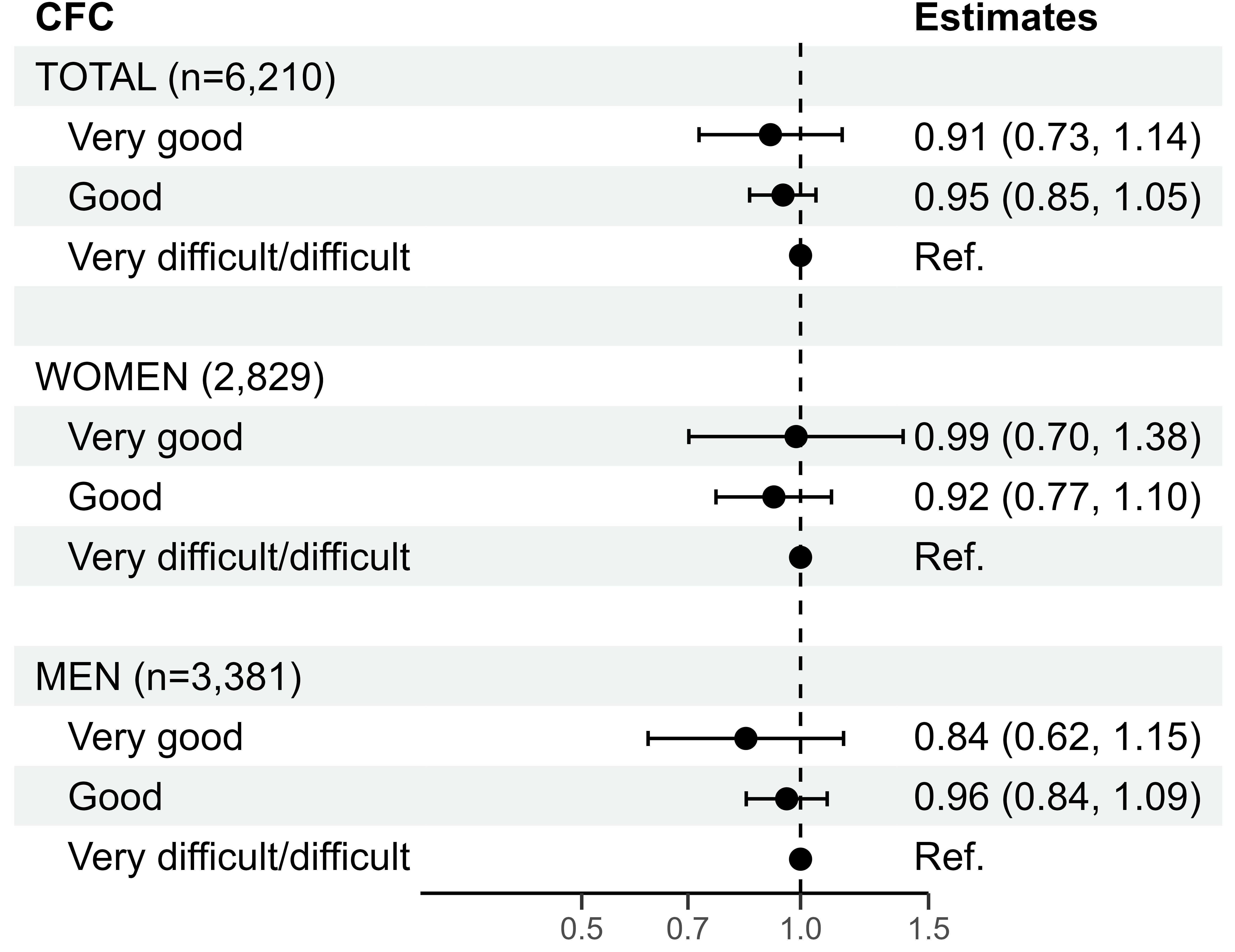
**

**Supplementary Figure S3**. Hazard ratios with 95% confidence intervals from Cox proportional hazards models for all-cause mortality at age 50 and older by childhood financial conditions (CFC).

Hazard ratios adjusted for age (timescale) and year of birth; total sample additionally adjusted for sex. Source: The Tromsø Study 1974–2022.

**Supplementary Table S2.** Odds ratios with 95% confidence intervals from logistic regression models for prevalence of chronic diseases at age 50–80 by highest educational level.

|  | **Cardiovascular diseases** | **Cancer** | **Diabetes** | **Chronic respiratory diseases** |
| --- | --- | --- | --- | --- |
| **Highest educational level** |  |  |  |  |
| **TOTAL** (n=4,567) |  |  |  |  |
| Long tertiary education | 0.56 (0.40, 0.78) | 1.50 (1.15, 1.96) | 0.53 (0.38, 0.73) | 0.56 (0.43, 0.73) |
| Short tertiary education | 0.75 (0.56, 1.01) | 1.45 (1.10, 1.91) | 0.70 (0.51, 0.94) | 0.69 (0.53, 0.90) |
| Upper secondary education | 1.02 (0.82, 1.27) | 1.19 (0.95, 1.50) | 0.81 (0.64, 1.01) | 0.74 (0.61, 0.89) |
| Primary education | Ref. | Ref. | Ref. | Ref. |
|  |  |  |  |  |
| **WOMEN** (n=2,066) |  |  |  |  |
| Long tertiary education | 0.45 (0.22, 0.93) | 1.75 (1.17, 2.60) | 0.43 (0.25, 0.75) | 0.47 (0.33, 0.69) |
| Short tertiary education | 1.25 (0.70, 2.23) | 1.66 (1.05, 2.64) | 0.79 (0.47, 1.35) | 0.56 (0.37, 0.85) |
| Upper secondary education | 1.01 (0.66, 1.56) | 1.44 (1.02, 2.05) | 0.88 (0.62, 1.27) | 0.74 (0.57, 0.97) |
| Primary education | Ref. | Ref. | Ref. | Ref. |
|  |  |  |  |  |
| **MEN** (n=2,501) |  |  |  |  |
| Long tertiary education | 0.59 (0.41, 0.86) | 1.30 (0.90, 1.87) | 0.60 (0.40, 0.89) | 0.66 (0.46, 0.96) |
| Short tertiary education | 0.65 (0.46, 0.92) | 1.28 (0.91, 1.81) | 0.65 (0.45, 0.95) | 0.79 (0.57, 1.10) |
| Upper secondary education | 1.02 (0.79, 1.31) | 1.00 (0.74, 1.35) | 0.76 (0.57, 1.02) | 0.72 (0.55, 0.95) |
| Primary education | Ref. | Ref. | Ref. | Ref. |

Odds ratios adjusted for age and year of survey; total sample additionally adjusted for sex. Source: The Tromsø Study 1994–2016.

**Supplementary Table S3.** Odds ratios with 95% confidence intervals from logistic regression models for prevalence of chronic diseases at age 50–80 by childhood financial conditions.

|  | **Cardiovascular diseases** | **Cancer** | **Diabetes** | **Chronic respiratory diseases** |
| --- | --- | --- | --- | --- |
| **Childhood financial conditions** |  |  |  |  |
| **TOTAL** (n=4,543) |  |  |  |  |
| Very good | 0.96 (0.63, 1.46) | 0.71 (0.45, 1.13) | 0.85 (0.55, 1.32) | 0.77 (0.53, 1.11) |
| Good | 0.90 (0.73, 1.09) | 1.06 (0.87, 1.30) | 0.91 (0.74, 1.12) | 0.82 (0.69, 0.97) |
| Very difficult/difficult | Ref. | Ref. | Ref. | Ref. |
|  |  |  |  |  |
| **WOMEN** (n=2,056) |  |  |  |  |
| Very good | 1.13 (0.58, 2.23) | 0.72 (0.37, 1.42) | 0.52 (0.25, 1.08) | 0.93 (0.58, 1.49) |
| Good | 0.71 (0.48, 1.04) | 1.04 (0.76, 1.43) | 0.69 (0.50, 0.95) | 0.88 (0.69, 1.13) |
| Very difficult/difficult | Ref. | Ref. | Ref. | Ref. |
|  |  |  |  |  |
| **MEN** (n=2,487) |  |  |  |  |
| Very good | 0.84 (0.50, 1.43) | 0.69 (0.37, 1.32) | 1.17 (0.67, 2.03) | 0.60 (0.34, 1.08) |
| Good | 0.97 (0.77, 1.22) | 1.08 (0.83, 1.40) | 1.09 (0.83, 1.42) | 0.77 (0.61, 0.98) |
| Very difficult/difficult | Ref. | Ref. | Ref. | Ref. |

Odds ratios adjusted for age and year of survey; total sample additionally adjusted for sex. Source: The Tromsø Study 1994–2016.


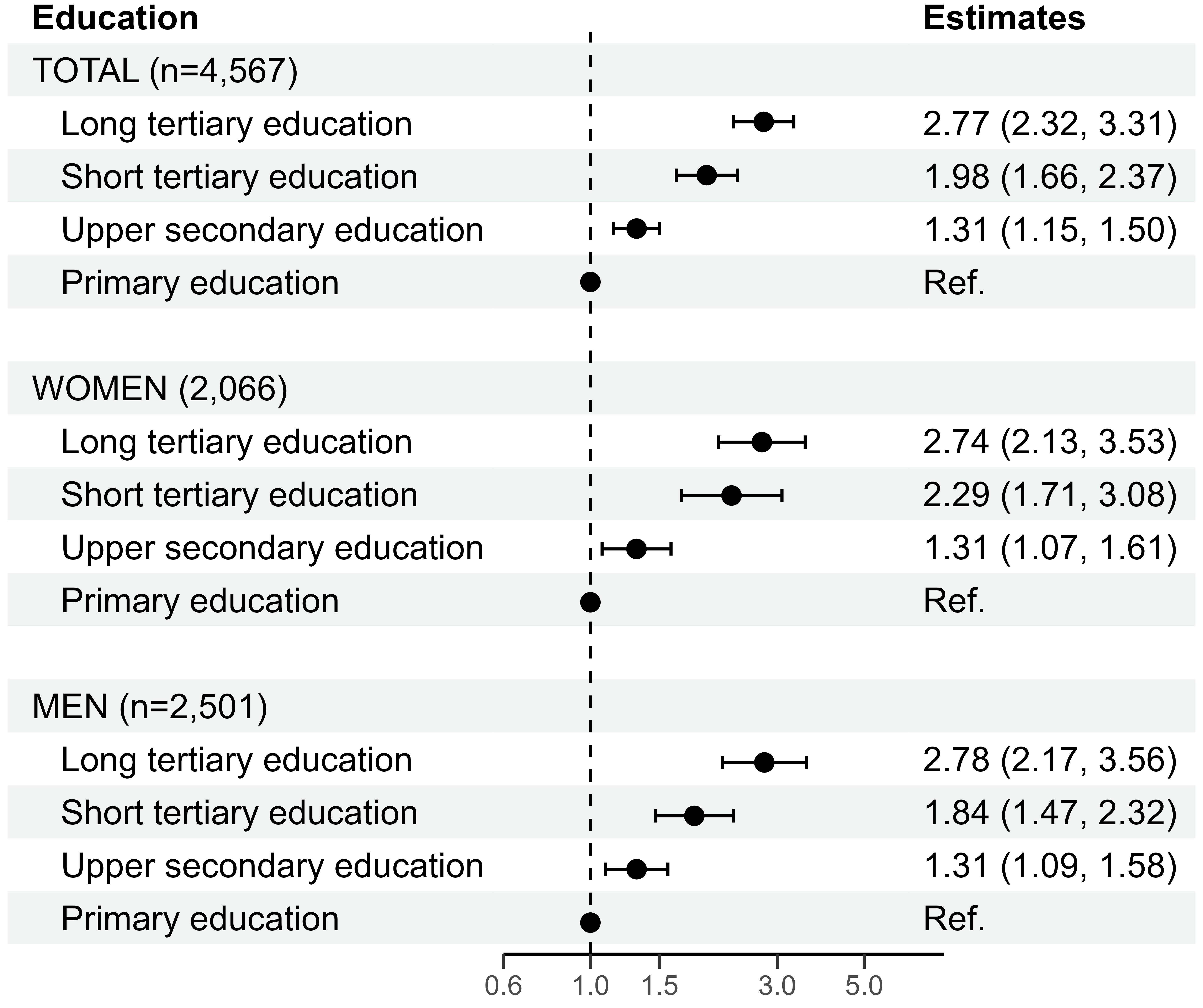


**Supplementary Figure S4.** Odds ratios with 95% confidence intervals from ordinal logistic regression models for self-rated health at age 50–80 by highest educational level.

Odds ratios adjusted for age and year of survey; total sample additionally adjusted for sex. Source: The Tromsø Study 1994–2016.


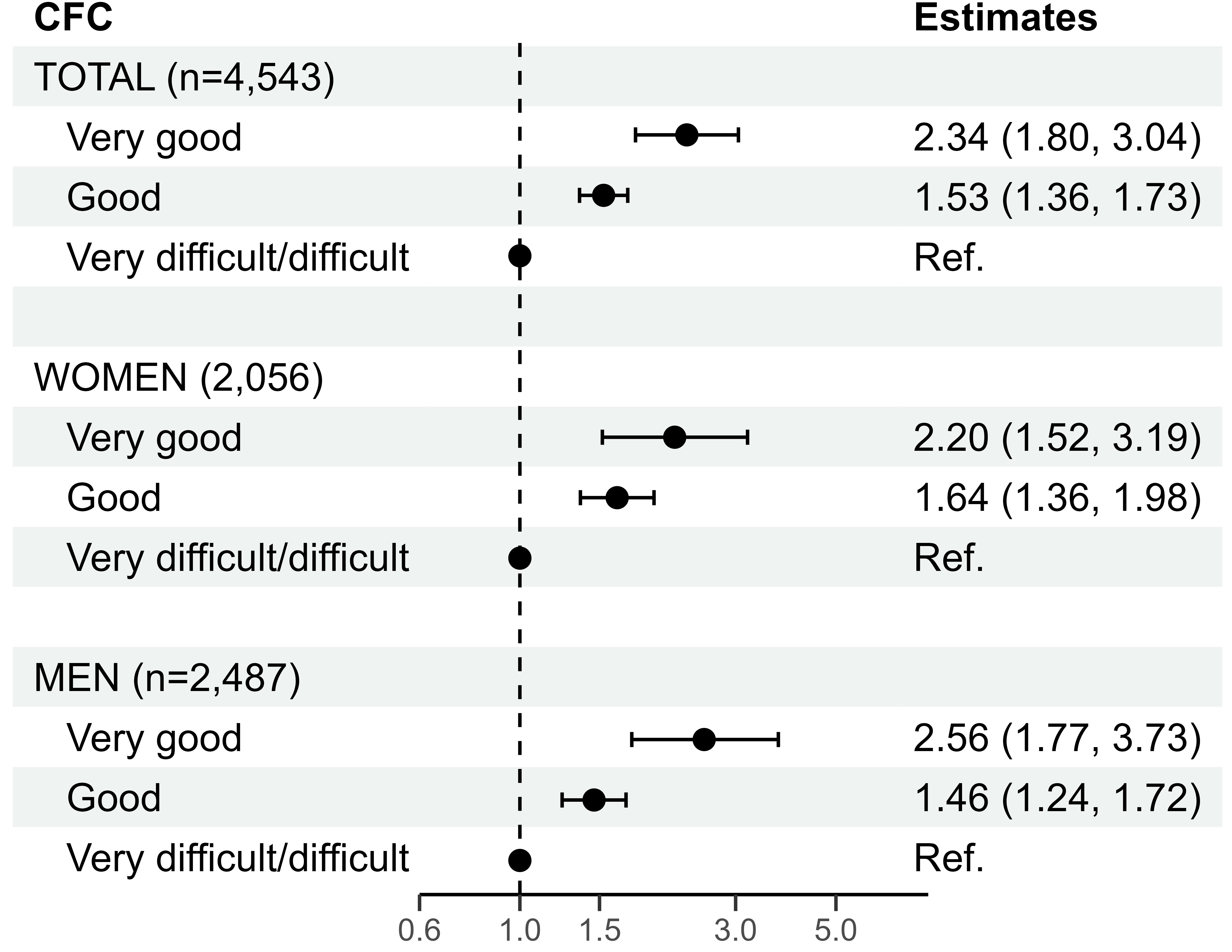


**Supplementary Figure S5.** Odds ratios with 95% confidence intervals from ordinal logistic regression models for self-rated health at age 50–80 by childhood financial conditions (CFC).

Odds ratios adjusted for age and year of survey; total sample additionally adjusted for sex. Source: The Tromsø Study 1994–2016.

**Supplementary Table S4.** Odds ratios with 95% confidence intervals from logistic regression models for prevalence of chronic diseases at age 50–80 by childhood socioeconomic position (CSEP) adjusted for highest educational level.

|  | **Cardiovascular diseases** | **Cancer** | **Diabetes** | **Chronic respiratory diseases** |
| --- | --- | --- | --- | --- |
| **TOTAL** (n=4,567) |  |  |  |  |
| *CSEP* |  |  |  |  |
| Non-manual | 0.87 (0.67, 1.14) | 1.11 (0.87, 1.42) | 1.15 (0.88, 1.49) | 0.78 (0.62, 0.98) |
| Skilled manual | 0.98 (0.73, 1.33) | 1.12 (0.84, 1.48) | 0.74 (0.53, 1.04) | 0.97 (0.76, 1.25) |
| Farmers | 1.14 (0.89, 1.45) | 0.95 (0.74, 1.23) | 1.23 (0.96, 1.57) | 0.85 (0.68, 1.05) |
| Unskilled/lower-skilled manual | Ref. | Ref. | Ref. | Ref. |
|  |  |  |  |  |
| *Highest educational level* |  |  |  |  |
| Long tertiary education | 0.59 (0.42, 0.84) | 1.43 (1.08, 1.90) | 0.52 (0.37, 0.73) | 0.60 (0.46, 0.79) |
| Short tertiary education | 0.77 (0.57, 1.05) | 1.41 (1.06, 1.86) | 0.70 (0.51, 0.95) | 0.72 (0.55, 0.94) |
| Upper secondary education | 1.04 (0.84, 1.30) | 1.17 (0.92, 1.47) | 0.82 (0.65, 1.03) | 0.75 (0.62, 0.91) |
| Primary education | Ref. | Ref. | Ref. | Ref. |
|  |  |  |  |  |
| **WOMEN** (n=2,066) |  |  |  |  |
| *CSEP* |  |  |  |  |
| Non-manual | 0.83 (0.49, 1.40) | 1.34 (0.93, 1.93) | 0.92 (0.60, 1.42) | 0.85 (0.61, 1.17) |
| Skilled manual | 1.01 (0.57, 1.78) | 1.03 (0.66, 1.61) | 0.66 (0.38, 1.14) | 1.06 (0.75, 1.49) |
| Farmers | 1.08 (0.67, 1.72) | 1.14 (0.76, 1.69) | 1.23 (0.84, 1.81) | 0.98 (0.72, 1.33) |
| Unskilled/lower-skilled manual | Ref. | Ref. | Ref. | Ref. |
|  |  |  |  |  |
| *Highest educational level* |  |  |  |  |
| Long tertiary education | 0.49 (0.23, 1.02) | 1.58 (1.03, 2.42) | 0.46 (0.26, 0.82) | 0.50 (0.34, 0.73) |
| Short tertiary education | 1.34 (0.74, 2.44) | 1.52 (0.94, 2.46) | 0.83 (0.48, 1.43) | 0.59 (0.38, 0.90) |
| Upper secondary education | 1.05 (0.67, 1.63) | 1.39 (0.97, 1.98) | 0.93 (0.64, 1.34) | 0.76 (0.58, 1.00) |
| Primary education | Ref. | Ref. | Ref. | Ref. |
|  |  |  |  |  |
| **MEN** (n=2,501) |  |  |  |  |
| *CSEP* |  |  |  |  |
| Non-manual | 0.87 (0.64, 1.19) | 0.96 (0.69, 1.34) | 1.29 (0.93, 1.80) | 0.73 (0.53, 1.01) |
| Skilled manual | 0.98 (0.69, 1.40) | 1.19 (0.82, 1.73) | 0.80 (0.52, 1.24) | 0.89 (0.62, 1.28) |
| Farmers | 1.16 (0.88, 1.53) | 0.85 (0.60, 1.18) | 1.23 (0.90, 1.69) | 0.73 (0.54, 1.00) |
| Unskilled/lower-skilled manual | Ref. | Ref. | Ref. | Ref. |
|  |  |  |  |  |
| *Highest educational level* |  |  |  |  |
| Long tertiary education | 0.63 (0.43, 0.94) | 1.28 (0.87, 1.88) | 0.56 (0.36, 0.85) | 0.73 (0.49, 1.07) |
| Short tertiary education | 0.67 (0.47, 0.95) | 1.26 (0.89, 1.78) | 0.65 (0.44, 0.95) | 0.82 (0.58, 1.14) |
| Upper secondary education | 1.05 (0.81, 1.35) | 0.99 (0.73, 1.34) | 0.76 (0.57, 1.02) | 0.74 (0.56, 0.97) |
| Primary education | Ref. | Ref. | Ref. | Ref. |

Odds ratios additionally adjusted for age and year of survey; total sample also adjusted for sex. Source: The Tromsø Study 1994–2016; occupational data from the Historical Population Register of Norway (original sources at the National Archives of Norway).

**Supplementary Table S5.** Hazard ratios with 95% confidence intervals from Cox proportional hazards models for all-cause mortality at age 50 and older by childhood socioeconomic position (CSEP) adjusted for highest educational level.

|  | **All-cause mortality** | |
| --- | --- | --- |
| **TOTAL** (n=6,636) | |  |
| *CSEP* | |  |
| Upper non-manual | | 1.15 (0.84, 1.58) |
| Lower non-manual | | 1.11 (0.97, 1.27) |
| Skilled manual | | 1.11 (0.96, 1.28) |
| Farmers | | 1.03 (0.91, 1.17) |
| Unskilled/lower-skilled manual | | Ref. |
|  | |  |
| *Highest educational level* | |  |
| Long tertiary education | | 0.49 (0.41, 0.59) |
| Short tertiary education | | 0.67 (0.57, 0.79) |
| Upper secondary education | | 0.94 (0.84, 1.05) |
| Primary education | | Ref. |
|  | |  |
| **WOMEN** (n=2,979) | |  |
| *CSEP* | |  |
| Upper non-manual | | 1.10 (0.58, 2.06) |
| Lower non-manual | | 1.19 (0.95, 1.49) |
| Skilled manual | | 1.07 (0.83, 1.37) |
| Farmers | | 1.00 (0.81, 1.23) |
| Unskilled/lower-skilled manual | | Ref. |
|  | |  |
| *Highest educational level* | |  |
| Long tertiary education | | 0.39 (0.28, 0.56) |
| Short tertiary education | | 0.62 (0.45, 0.86) |
| Upper secondary education | | 0.97 (0.81, 1.17) |
| Primary education | | Ref. |
|  | |  |
| **MEN** (n=3,657) | |  |
| *CSEP* | |  |
| Upper non-manual | | 1.17 (0.81, 1.69) |
| Lower non-manual | | 1.08 (0.91, 1.27) |
| Skilled manual | | 1.13 (0.95, 1.36) |
| Farmers | | 1.05 (0.90, 1.23) |
| Unskilled/lower-skilled manual | | Ref. |
|  | |  |
| *Highest educational level* | |  |
| Long tertiary education | | 0.53 (0.43, 0.66) |
| Short tertiary education | | 0.68 (0.56, 0.83) |
| Upper secondary education | | 0.91 (0.80, 1.05) |
| Primary education | | Ref. |

Hazard ratios additionally adjusted for age (timescale) and year of birth; total sample also adjusted for sex. Source: The Tromsø Study 1974–2022; occupational data from the Historical Population Register of Norway (original sources at the National Archives of Norway).**Supplementary Table S6.** Odds ratios with 95% confidence intervals from ordinal logistic regression models for self-rated health at age 50–80 by childhood socioeconomic position (CSEP) adjusted for highest educational level.

|  | **Self-rated health** | |
| --- | --- | --- |
| **TOTAL** (n=4,567) | |  |
| *CSEP* | |  |
| Upper non-manual | | 1.52 (1.06, 2.19) |
| Lower non-manual | | 1.16 (0.99, 1.36) |
| Skilled manual | | 1.08 (0.91, 1.29) |
| Farmers | | 0.91 (0.78, 1.06) |
| Unskilled/lower-skilled manual | | Ref. |
|  | |  |
| *Highest educational level* | |  |
| Long tertiary education | | 2.51 (2.08, 3.03) |
| Short tertiary education | | 1.89 (1.57, 2.26) |
| Upper secondary education | | 1.27 (1.11, 1.46) |
| Primary education | | Ref. |
|  | |  |
| **WOMEN** (n=2,066) | |  |
| *CSEP* | |  |
| Upper non-manual | | 2.17 (1.27, 3.71) |
| Lower non-manual | | 1.24 (0.98, 1.57) |
| Skilled manual | | 1.17 (0.90, 1.51) |
| Farmers | | 0.87 (0.69, 1.10) |
| Unskilled/lower-skilled manual | | Ref. |
|  | |  |
| *Highest educational level* | |  |
| Long tertiary education | | 2.32 (1.77, 3.04) |
| Short tertiary education | | 2.08 (1.54, 2.82) |
| Upper secondary education | | 1.24 (1.01, 1.53) |
| Primary education | | Ref. |
|  | |  |
| **MEN** (n=2,501) | |  |
| *CSEP* | |  |
| Upper non-manual | | 1.12 (0.69, 1.82) |
| Lower non-manual | | 1.09 (0.87, 1.35) |
| Skilled manual | | 1.02 (0.80, 1.30) |
| Farmers | | 0.94 (0.77, 1.16) |
| Unskilled/lower-skilled manual | | Ref. |
|  | |  |
| *Highest educational level* | |  |
| Long tertiary education | | 2.67 (2.06, 3.47) |
| Short tertiary education | | 1.81 (1.44, 2.28) |
| Upper secondary education | | 1.30 (1.08, 1.56) |
| Primary education | | Ref. |

Odds ratios additionally adjusted for age and year of survey; total sample also adjusted for sex. Source: The Tromsø Study 1994–2016; occupational data from the Historical Population Register of Norway (original sources at the National Archives of Norway).
